# A study demonstrating users’ preference for the adapted-REQUITE patient-reported outcome questionnaire over PRO-CTCAE^®^ in patients with lung cancer

**DOI:** 10.3389/fonc.2024.1328871

**Published:** 2024-04-10

**Authors:** Thomas Jordan, Thitikorn Nuamek, Isabella Fornacon-Wood, Raffaele Califano, Joanna Coote, Margaret Harris, Hitesh Mistry, Paul Taylor, David Woolf, Corinne Faivre-Finn

**Affiliations:** ^1^ Division of Cancer Sciences, Faculty of Biology, Medicine, and Health, University of Manchester, Manchester, United Kingdom; ^2^ The Christie NHS Foundation Trust, Manchester, United Kingdom; ^3^ Wrightington, Wigan and Leigh Teaching Hospitals NHS Foundation Trust, Wigan, United Kingdom

**Keywords:** patient reported outcome, CTCAE, REQUITE, quality of life, symptoms, lung cancer

## Abstract

**Introduction:**

The use of patient-reported outcomes (PROs) has been shown to enhance the accuracy of symptom collection and improve overall survival and quality of life. This is the first study comparing concordance and patient preference for two PRO tools: Patient-Reported Outcomes version of the Common Terminology Criteria for Adverse Events (PRO-CTCAE^®^) and the adapted-REQUITE Lung Questionnaire.

**Materials and Methods:**

Patients with lung cancer were recruited to the study while attending outpatient clinics at a tertiary cancer centre. Clinician-reported outcomes were generated through initial patient assessment with CTCAE v4.03. Participants then completed the PRO-CTCAE^®^ and adapted-REQUITE questionnaires. Concordance between the 2 questionnaires was assessed by calculating Pearson correlation coefficient. PRO-CTCAE^®^ and CTCAE concordance was demonstrated by calculating Pearson correlation coefficient from the linear predictors of an ordinal logistic regression. P-values were also calculated.

**Results:**

Out of 74 patients approached, 65 provided written informed consent to participate in the study. 63 (96.9%) patients completed both PRO-CTCAE^®^ and adapted-REQUITE questionnaires. Pearson correlation coefficient between PRO tools was 0.8-0.83 (p <.001). Correlation between CTCAE and PRO-CTCAE^®^ ranged between 0.66-0.82 (p <.001). Adapted-REQUITE and CTCAE correlation was higher for all symptoms ranging between 0.79-0.91 (p <.001). Acceptable discrepancies within one grade were present in 96.8%-100% of symptom domains for REQUITE and in 92.1%-96.8% for all domains in the PRO-CTCAE^®^. 54% of the total participant cohort favored the adapted-REQUITE questionnaire due to reduced subjectivity in the questions and ease of use.

**Conclusion:**

The adapted-REQUITE questionnaire has shown a superior correlation to clinician-reported outcomes and higher patient preference than the PRO-CTCAE^®^. The results of this study suggest the use of the REQUITE questionnaire for patients with lung cancer in routine clinical practice.

## 1 Introduction

Lung cancer is one of the most common cancers worldwide, with an incidence rate of 2.5 million cases in 2022 (1). Despite advances in cancer treatment and increased smoking cessation efforts, lung cancer remains the leading cause of cancer death, and its 10-year survival rate remains below 10% (2).

Treatment for lung cancer varies depending on the stage and type of cancer, and all treatment regimens are associated with significant side-effects (3). For example, platinum-based chemotherapy, used in patients with locally advanced and metastatic lung cancer causes significant side effects such as fatigue, peripheral neuropathy, and nausea/vomiting, and can have a negative impact on quality of life (4). For patients receiving radiotherapy, the most associated dose-limiting toxicity is radiation pneumonitis which is predicted to affect between 13-37% of patients (5, 6). Surgery is also an important treatment option, particularly for those patients with early-stage lung cancer. Common complications include lung collapse, bleeding, irregular heartbeat, infections at the surgical site or in the lungs, air leakage from the lungs and difficulty breathing (7–10).

Adequate assessment and reporting of patient symptoms and adverse events are required to manage patients with cancer effectively. Assessment of symptoms is especially important for patients with lung cancer, as research suggests up to 75% of lung cancer patients will experience symptomatic disease progression (11). Traditionally, clinicians monitor patients for side effects and manage adverse events (AEs) based on their perceived severity. The current gold standard for reporting AEs, particularly in the context of clinical trials, is the National Cancer Institute (NCI)’s Common Terminology Criteria for Adverse Events (CTCAE) (12). It features a bank of 790 items and provides a severity grading scale from 1 to 5. Due to its comprehensive nature, the CTCAE is used in routine clinical practice for patient symptom assessment since its inception in 1983 (13).

The use of the CTCAE has numerous limitations. A large body of evidence shows that clinician-reported AEs may miss or underreport up to 50% of symptoms that patients experience during treatment (14–19). Discrepancies between patient and physician reporting tend to be satisfactory for objectively measurable symptoms such as vomiting; however, large discrepancies exist in subjective symptoms such as nausea and fatigue (16). Therefore, there is an unmet need to incorporate patients’ perspectives into AE reporting.

Over the last decade, patient-reported outcomes (PROs) have found increased utility in clinical trials, and there is an increased effort to incorporate PROs into routine clinical practice. Large-scale randomized controlled trials have reported that incorporating PROs into clinical practice decreases hospital admission, improves anti-cancer therapy compliance, improves quality of life, and increases overall survival (20–23). Furthermore, PROs have been shown to guide follow-up consultations, allowing comprehensive management of patients, mental status, cancer, and adverse events from treatment (24). The collection of PROs has also been described by the Food and Drug Administration (FDA) and the European Society of Medical Oncology (ESMO) as the gold standard of symptom collection (25, 26).

Noting the need to incorporate PROs into symptom assessment, NCI developed a patient-reported version of the CTCAE (PRO-CTCAE^®^). The PRO-CTCAE^®^ questionnaire is a library of 124 questions evaluating 78 toxicities mapped to the CTCAE (27). The questionnaire features up to three questions per AE item, assessing different symptom attributes: frequency, severity, interference, amount, and presence/absence. The PRO-CTCAE^®^ uses a 5-level severity grading scale from 0 to 4, except for the presence/absence attribute which uses 0 to 1. The PRO-CTCAE^®^ questionnaire has proven utility in a large multi-national clinical trial (28).

Another PRO tool that was integrated in a multi-national study is the questionnaire developed as part of the European Union funded REQUITE study (29, 30). It features a direct lay translation of the CTCAE for symptoms commonly experienced during lung cancer radiotherapy and chemotherapy. Symptoms are graded based on severity from 0 to 4. The REQUITE questionnaire has been formally validated at The Christie NHS Foundation Trust (31). By way of example, **Table 1** compares how CTCAE, PRO-CTCAE^®^ and REQUITE grade the severity of anorexia (27, 29, 32).

**Table 1:** Reporting difference between CTCAE, PRO-CTCAE^®^ and REQUITE for anorexia.

Grade	CTCAE v4.03 Anorexia	PRO-CTCAE^®^ In the last 7 days, what was the SEVERITY of your DECREASED APPETITE at its WORST?	PRO-CTCAE^®^In the last 7 days, how much did DECREASED APPETITE INTERFERE with your usual or daily activities	REQUITE Have you lost your appetite?
**0**	None	None	Not at all	I have not lost my appetite
**1**	Loss of appetite without alteration in eating habits.	Mild	A little bit	I have lost my appetite, but I eat as I normally would
**2**	Oral intake altered without significant weight loss or malnutrition; oral nutritional supplements indicated.	Moderate	Somewhat	I have lost my appetite and lost weight. I have been prescribed food supplements.
**3**	Associated with significant weight loss or malnutrition (e.g., inadequate oral caloric and/or fluid intake); tube feeding or TPN indicated.	Severe	Quite a bit	I have lost my appetite and lost weight. I have been getting extra fluids or nutrition with a tube or a drip.
**4**	Life threatening consequences; urgent intervention indicated.	Very Severe	Very much	I have been in hospital with severe/life-threatening weight loss.

CTCAE, Common Terminology Criteria for Adverse Events; PRO-CTCAE^®^, Patient-Reported Outcomes version of Common Terminology Criteria for Adverse Events; TPN, Total parental nutrition.

Both PRO-CTCAE^®^ and adapted-REQUITE questionnaires have strengths and limitations. The PRO-CTCAE^®^ severity grading scale offers a subjective grading approach open to interpretation. On the other hand, the REQUITE questionnaire features a direct lay translation of CTCAE and detailed, objective descriptions for each grade. There is therefore an unmet need to compare existing PRO tools and assess patients’ preference in the context of routine clinical practice. This study compared the PRO-CTCAE^®^ and REQUITE PRO tools in conjunction with clinician-reported outcomes and explored patient preference through surveys. The aim was to identify the most appropriate questionnaire to use in our institution in a clinical practice setting.

## 2 Materials and methods

This was a single-site, prospective questionnaire-based study conducted at The Christie NHS Foundation Trust. Approval for this study was obtained from the Health Research Authority, London – City and East Research Ethics Committee, and The Christie Research and Development Division. To identify the most appropriate questionnaire to use at our institution, this study had three primary objectives: 1) to conduct correlation analyses between clinician-reported CTCAE and each PRO tool, 2) to assess the correlations between both PRO tools, and 3) to determine patient preference between these PRO tools.

Patients with lung cancer at outpatient clinics were approached and recruited to the study with written informed consent. Any individuals aged over 18 years who could provide informed consent with a confirmed histological or clinical diagnosis of lung cancer were included. All patients, with or without prior surgical invention, were either undergoing radiotherapy, chemo-radiotherapy, systemic anti-cancer treatment, or were in post-treatment follow-up. Those patients who could not read or understand English were not recruited into the study.

Upon obtaining written informed consent, symptoms were assessed verbally by a clinician using CTCAE v4.03, which consisted of 8 domains: performance status, shortness of breath, chest pain, dysphagia, cough, hemoptysis, reduced appetite, and fatigue. The Adult Comorbidity Score 27 (ACE-27) was additionally completed for all patients by their clinicians. Each patient was provided with two PRO tools to complete in a paper format: the PRO-CTCAE^®^ questionnaire and the adapted-REQUITE Lung questionnaire. The latter questionnaire was adapted from a questionnaire developed as part of the European Union funded REQUITE project (29). The adapted-REQUITE Lung questionnaire featured 8 questions assessing 8 domains: performance status, shortness of breath, chest pain, dysphagia, cough, hemoptysis, weight loss, and fatigue (See **Supplementary Material**). The PRO-CTCAE^®^ questionnaire included adverse events and symptoms commonly experienced by patients with lung cancer during treatment or follow-up. It consisted of 12 questions assessing 6 symptom domains: shortness of breath, chest pain, dysphagia, cough, reduced appetite, and fatigue (See **Supplementary Material**).

An evaluation questionnaire was also given to determine which PRO tool patients felt best described their symptoms, the ease of filling out the questionnaires, and their overall preference between the two PRO tools. Patient data (including age, gender, disease staging, histology, ECOG performance status, ACE-27 score, and smoking history) and treatment data were collected from structured forms included in the electronic patient records.

A power calculation was performed to determine the sample size required for the study using a conservative correlation coefficient of 0.3. The calculation set a target sample size of 86 patients. Data obtained from CTCAE and both PRO tools were assessed for its distribution by visual inspection. Correlation between CTCAE and adapted-REQUITE was calculated using the Pearson correlation coefficient for linear relationships between variables, which was reported together with the corresponding p-value. The correlation between CTCAE and PRO-CTCAE^®^ was assessed via an ordinal logistic regression analysis since one question on the CTCAE related to multiple questions on the PRO-CTCAE^®^. The linear predictor from the ordinal logistic regression analysis was used to calculate the Pearson correlation coefficient and corresponding p-value. Correlation analysis was only performed for symptoms at least 3 patients reported having. All analyses were conducted in R v3.4.1, and statistical significance was set at p-value <.05.

## 3 Results

### 3.1 Patient recruitment

**Figure 1:**
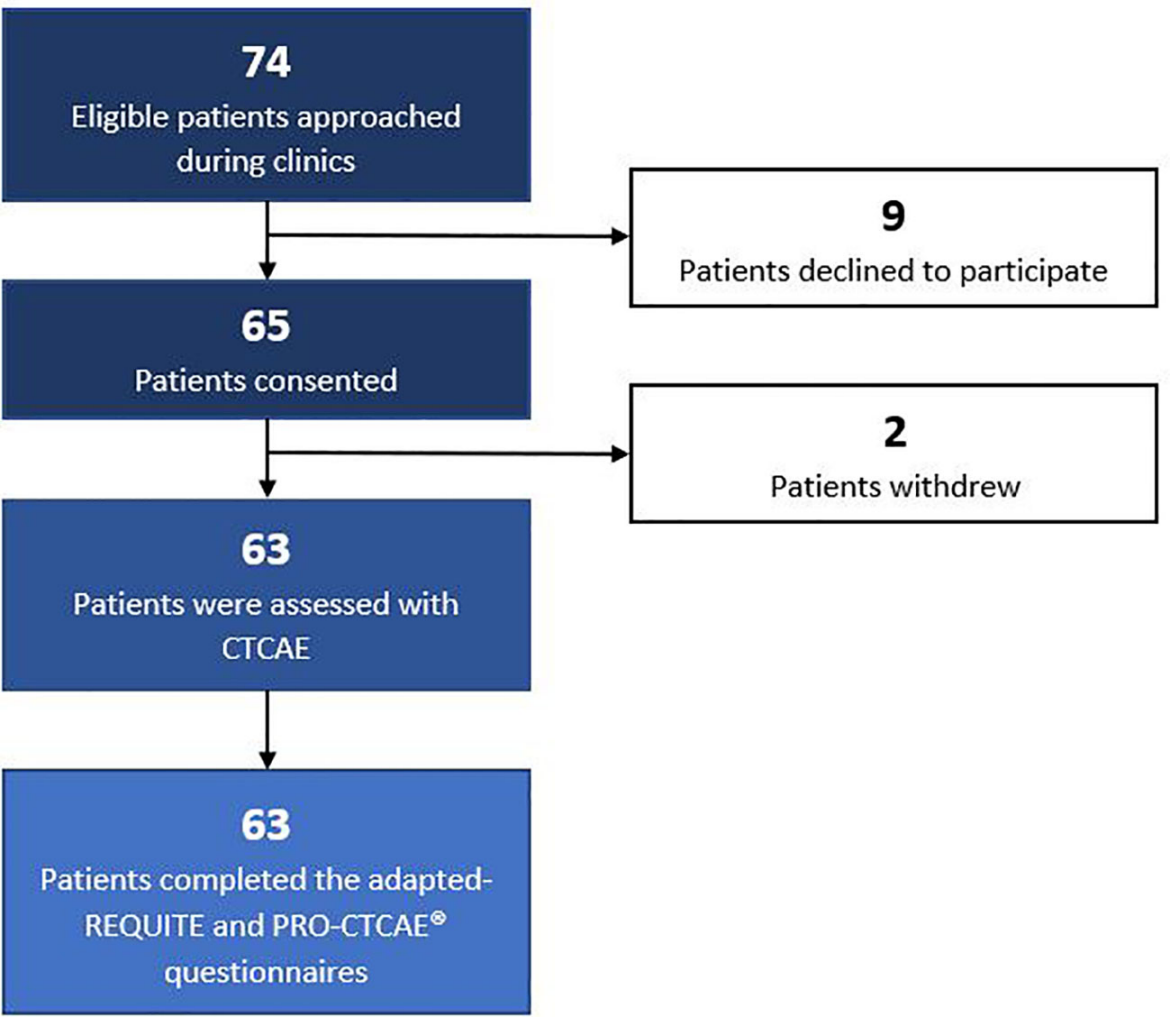
Patient recruitment flow.

Patients were recruited in June 2018. Out of 74 eligible patients approached to participate in the study, 9 declined to participate in the study. The reasons for declining were as follows: 4 patients reported anxiety related to their appointments, 3 patients mentioned time constraints, 1 patient expressed concern about questionnaire complexity, and 1 patient did not provide a specific reason. A total of 65 patients provided written informed consent to participate in the study. Subsequently, 2 patients withdrew their consent following their consultation, one due to receiving bad news and the other due to not receiving the requested pharmaceutical agent. Overall, the final study cohort comprised 63 patients. The recruitment workflow is depicted in **Figure 1**.

### 3.2 Patient characteristics

Patient characteristics are presented in **Table 2**. There was no missing data. The median age was 68 years, ranging from 47 to 89. Among the study population, 63.5% were female, and 57.1% were ex-smokers. The predominant diagnosis and disease stage was stage IV non-small cell lung cancer (NSCLC) (44.4%). Clinician-evaluated ECOG performance status ranged from 0 to 3, with the majority having a score of 1 (52.4%). As patients were recruited from different oncology clinics, patients underwent various types of cancer treatment. This was categorized as chemotherapy, radiotherapy, systemic therapy, which includes targeted therapies and immunotherapy agents, and post-treatment follow-up.

**Table 2:** Baseline patient characteristics.

	All patients (n=63)
**Age in years, median (range)**	68 (47–89)
**Gender, n (%) ** ** Male** ** Female**	23 (36.5) 40 (63.5)
**Diagnosis and staging ** ** NSCLC** ** I** ** II** ** III** ** IV** ** SCLC** ** Limited** ** Extensive**	51 (81.0) 10 (15.9) 3 (4.8) 10 (15.9) 28 (44.4) 12 (19.0) 6 (9.5) 6 (9.5)
**NSCLC histology ** ** Adenocarcinoma** ** Squamous cell carcinoma** ** Unknown**	37 (58.7) 9 (14.3) 5 (7.9)
**Smoking status ** ** Ex** ** Current** ** Never**	36 (57.1) 14 (22.2) 13 (20.6)
**Pack years ** ** <15** ** 15-30** ** 31-45** ** 46-60** ** >60** ** Unknown**	2 (3.2) 16 (25.4) 14 (22.2) 12 (19.0) 3 (4.8) 4 (6.3)
**ECOG Performance status ** ** 0** ** 1** ** 2** ** 3**	17 (27.0) 33 (52.4) 9 (14.3) 4 (6.3)
**ACE-27 ** ** 0** ** 1** ** 2** ** 3** ** Unknown**	14 (22.2) 23 (36.5) 11 (17.5) 7 (11.1) 8 (12.7)
**Patients on treatment ** ** Chemotherapy** ** Radiotherapy** ** Systematic therapy** **Patients on follow-up**	10 (15.9) 21 (33.3) 19 (30.2) 13 (20.6)

Systemic therapy included tyrosine kinase inhibitors and immunotherapy agents. NSCLC, Non-Small Cell Lung Cancer; SCLC, Small Cell Lung Cancer; ECOG: Eastern Cooperative Oncology Group; ACE-27, Adult Comorbidities Evaluation 27.

### 3.3 Correlations between CTCAE and PRO tools

The clinician-reported CTCAE symptom assessment and the patient-reported adapted-REQUITE and PRO-CTCAE^®^ symptom assessment are included in **Supplementary Tables 1**-**3**. The most common clinician-reported CTCAE symptoms of any grade were fatigue (68.3%), shortness of breath (65.1%) and cough (57.1%). Shortness of breath was the only grade 4 symptom reported. The most common patient-reported symptoms on the adapted-REQUITE questionnaire were fatigue (71.4%), shortness of breath (66.6%) and cough (57.1). Again, only one grade 4 symptom was reported, which was shortness of breath. The most common patient-reported symptoms on the PRO-CTCAE^®^ questionnaire were fatigue (69.8%), shortness of breath (65.1%) and cough (50.8%). Across six symptom domains, including dysphagia, chest pain, shortness of breath, cough, reduced appetite, and fatigue, a higher number of grade 3-4 severity were reported in PRO-CTCAE^®^ (8.99%) compared to adapted-REQUITE (1.84%).

**Supplementary Table 4** reports the grading differences between clinician-reported CTCAE and patient-reported adapted-REQUITE. Exact grade matching ranged from 69.8% (shortness of breath) to 84.1% (performance status). Discrepancies within one grade were present in 96.8% to 100% of symptom domains. For all domains, there were no discrepancies over two grade differences. **Supplementary Table 5** reports the grading differences between CTCAE and PRO-CTCAE^®^. Exact grade matching ranged from 49.2% (fatigue) to 85.7% (dysphagia). Discrepancies within one grade were present in 92.1% to 98.6% of symptom domains. For all domains, there were no discrepancies over two grade differences. Symptom grade agreement was higher between CTCAE and adapted-REQUITE (>69.8%) than PRO-CTCAE^®^ (>60.3%).

**Table 3** presents the correlation between CTCAE symptoms and symptoms reported through adapted-REQUITE or PRO-CTCAE^®^. Statistical analysis was conducted on symptoms reported by enough patients. The correlation between CTCAE and adapted-REQUITE symptoms ranged from 0.72 (reduced appetite) to 0.91 (performance status). The correlation between CTCAE and PRO-CTCAE^®^ symptoms ranged from 0.63 (reduced appetite) to 0.82 (shortness of breath). All CTCAE and PRO-tool symptoms had statistically significant positive correlations (p <.001).

**Table 3:** Correlation between CTCAE clinician-reported symptoms and adapted-REQUITE or PRO-CTCAE^®^ patient-reported symptoms.

Symptoms	CTCAE vs adapted REQUITE		CTCAE vs PRO-CTCAE^®^	
	Correlation (95% CI)	P-value	Correlation (95% CI)	P-value
**Performance Status**	0.91 (0.86, 0.94)	<.001		
**Shortness of Breath**	0.79 (0.67, 0.87)	<.001	0.82 (0.72, 0.89)	<.001
**Fatigue**	0.79 (0.67, 0.87)	<.001	0.66 (0.49, 0.78)	<.001
**Cough**	0.77 (0.65, 0.85)	<.001	0.73 (0.59, 0.83)	<.001
**Reduced appetite**	0.72 (0.57, 0.82)	<.001	0.63 (0.45, 0.76)	<.001

CTCAE, Common Terminology Criteria for Adverse Events; PRO-CTCAE^®^, Patient-Reported Outcomes version of Common Terminology Criteria for Adverse Events; CI, Confidence Interval.

### 3.4 Correlations between PRO-CTCAE^®^ and adapted-REQUITE

**Table 4** presents the correlation between two PRO tools, PRO-CTCAE^®^ and adapted-REQUITE. There was a high correlation between PRO-CTCAE^®^ and adapted-REQUITE symptoms (0.80-0.83, p <.001).

**Table 4:** Correlation between PRO-CTCAE^®^ and adapted-REQUITE patient-reported symptoms.

Symptoms	Correlation (95% CI)	P-value
**Shortness of breath**	0.82 (0.72, 0.89)	<.001
**Fatigue**	0.80 (0.69, 0.87)	<.001
**Cough**	0.83 (0.73, 0.89)	<.001
**Reduced appetite**	0.83 (0.73, 0.89)	<.001

PRO-CTCAE^®^, Patient-Reported Outcomes version of Common Terminology Criteria for Adverse Events; CI, Confidence Interval.

### 3.5 Patient preference

The results from the patient evaluation questionnaire are presented in **Table 5**. 51% of patients felt adapted-REQUITE better described their symptoms, compared to 11% for PRO-CTCAE. 38% found both questionnaires described their symptoms equally, although most of these patients scored zero in all domains in both questionnaires. Regarding ease of completion, 38% expressed no preference, while 37% and 25% found adapted-REQUITE and PRO-CTCAE^®^ easier to complete respectively. When asked which questionnaire patients preferred overall, more than half (54%) favored adapted-REQUITE, with 24% having no preference and 22% favoring PRO-CTCAE. The reasons patients prefer adapted-REQUITE or PRO-CTCAE^®^ are included in **Supplementary Tables 6**, **7**. The most common reason for preferring adapted-REQUITE was that it better described their symptoms while for PRO-CTCAE^®^ was that it was easier to complete.

**Table 5:** Patient evaluation questionnaire.

	Adapted-REQUITE	PRO-CTCAE^®^	No preference
**Which of the two PRO tools best describes your symptoms?**	51%	11%	38%
**Which of the two questionnaires did you find easiest to complete?**	37%	25%	38%
**Overall which questionnaire did you prefer?**	54%	22%	24%

PRO-CTCAE, Patient-Reported Outcomes version of Common Terminology Criteria for Adverse Events.

## 4 Discussion

This study has several key findings. Firstly, both PRO tools showed a strong correlation with clinician-reported outcomes using CTCAE, with the adapted-REQUITE questionnaire showing a higher correlation than the PRO-CTCAE^®^ questionnaire. Secondly, the adapted-REQUITE questionnaire also showed higher levels of exact grading agreement and agreement within one grade up to 100%. Thirdly, most patients expressed an overall preference for adapted-REQUITE over PRO-CTCAE^®^ as more patients found the adapted-REQUITE questionnaire better described their symptoms.

To our knowledge, this is the first study comparing the PRO-CTCAE^®^ and adapted-REQUITE PRO tools for concordance and overall patient preference in a lung cancer population. Both PRO tools have been previously validated and have proven utility in the clinical environment (31, 33, 34). This study further demonstrates the feasibility of incorporating PRO tools in routine clinical practice. Overall, both PRO questionnaires were received very well by the patients and their clinicians, as the study had a high uptake of participants. The two PRO tools were completed by all 63 patients, and an average of 21 patients were recruited per week. Only 9 patients refused to participate, and 2 patients withdrew their consent due to external factors unrelated to the study.

In this study, a strong correlation was observed between clinician-reported CTCAE and patient responses to both PRO tools. The correlation was statistically significant with p <.001 for shortness of breath, fatigue, cough, and reduced appetite. Pearson rho values range from 0.66 to 0.82 for PRO-CTCAE^®^ and 0.79 to 0.91 for the adapted-REQUITE questionnaire. Furthermore, the agreement between each PRO tool and clinician-reported symptoms using CTCAE was also high. The PRO-CTCAE^®^ questionnaire had an exact grading agreement of 49.2% to 85.7%, and agreement within one grade ranged from 92.1% to 98.4%. The adapted-REQUITE questionnaire demonstrated a higher exact grading agreement for symptoms of 69.8% to 84.1%, with agreement within one grade ranging from 96.8% to 100%. Importantly, neither questionnaire displayed discrepancies higher than two grades. This study’s findings align with existing literature that discrepancies between patient and clinician reporting are notably higher for subjective symptoms (16). The weakest correlation for both PRO tools was observed for fatigue and reduced appetite. However, even for these symptoms, discrepancies within one grade remained above ninety percent in both questionnaires.

As already mentioned, the correlation levels observed between CTCAE and both PRO tools were high. However, the findings of this study suggest that the adapted-REQUITE questionnaire has a superior correlation to CTCAE than the PRO-CTCAE^®^ in most variable domains, except shortness of breath. Therefore, the adapted-REQUITE questionnaire is superior to the PRO-CTCAE^®^ in this study. It should be noted that the study was powered with a conservative correlation of 0.3, generating a target size of 86 patients. The trial sponsorship was significantly delayed due to factors beyond the control of the study investigator; as a result, recruitment time was cut to three weeks, limiting the total number of patients that could be recruited. However, the correlation between the PRO tools was significantly higher than the correlation used in the power calculation, and the total of 63 recruited participants was sufficient for statistical analysis.

The correlation between the clinician-reported outcomes and PRO tools in this study surpasses the correlations reported in other studies. For instance, the correlation for fatigue was 0.66 with the PRO-CTCAE^®^ questionnaire and 0.79 with adapted-REQUITE. For shortness of breath, the values were reported as 0.82 and 0.79 for the PRO-CTCAE^®^ and adapted-REQUITE questionnaires respectively. A 2006 study by Basch et al. involving 400 patients found a correlation of 0.55 for fatigue between clinician-reported CTCAE and patient-reported syntactically modified CTCAE (15). Another study reported a correlation for fatigue as low as 0.3 between clinician-reported CTCAE and the European Organization for Research and Treatment Cancer’s Quality of Life questionnaire (EORTC QLQ-C30) completed by patients (35). When comparing PRO-CTCAE^®^ to EORTC QLQ-C30, there was a correlation of 0.74 for fatigue and 0.47 for shortness of breath (34). In our study, there was no time limit for questioning patients and determining CTCAE grading. The study also took place in a private room separate from busy lung cancer clinics. Therefore, the factors of time pressures and busy clinic environment that traditionally affect clinician reporting may have been absent here.

As the level of correlation between the two PRO tools is very high, patient preference is a significant factor when determining overall superiority. Patient preference was broken down into three categories: those that preferred the adapted-REQUITE questionnaire (54%), PRO-CTCAE^®^ (22%), and those that stated the tools were equal (24%). The largest group of participants stated that they preferred the adapted-REQUITE questionnaires overall. The most commonly stated reason was that the descriptions of the grades removed the subjectivity present in the none-to-severe grading of the PRO-CTCAE^®^. One patient who had suffered from significant dysphagia stated that grading subjectively would require interpretation by clinicians as his response did not sit accurately in one domain. Such situations may create some subjectivity and lead to clinicians under- or overestimating the severity of patient symptoms. A minority of 22% of patients stated they preferred the PRO-CTCAE^®^. The most common reason people stated for PRO-CTCAE^®^ preference was that the subjective approach was more straightforward to complete. It should be considered that PRO tools will often be completed during a time of increased anxiety; therefore, simplicity and ease of completion are significant factors for patient preference. The questionnaires for this study were completed in the clinical environment, and some patients were particularly anxious about their scan results.

There are some limitations in this study. It was a single-site study with a limited yet diverse patient population. The inclusion of individuals undergoing different treatments and at various stages of their disease introduces variability to the findings; the number of patients reporting certain symptoms such as chest pain, dysphagia, and hemoptysis was insufficient for analysis, underscoring the need for further research to establish correlations for these symptoms. Notably, the study participants were relatively younger, with a median age of 68, while more than half of patients diagnosed with lung cancer are 70 years and above (2). Additionally, the proportion of female patients was larger than the national estimates (63.5% in our study versus 48% nationally). Finally, deprivation index data was not available in this study and we therefore cannot confirm the diversity of socio-economic backgrounds.

Several participants required assistance to complete the questionnaires. The adapted-REQUITE questionnaire is shorter than the PRO-CTCAE^®^ in terms of the number of questions. However, the volume of reading required meant that some participants still needed assistance from their families or the study investigator to complete the questionnaire. The main issue was that the grade descriptions were quite long; therefore, some patients struggled due to poor literacy skills or eyesight. For 7 patients, the study investigator had to read the questionnaire to the patient. Approximately 5 patients required help from their relatives either to read or to interpret the questionnaire; however, the exact number, although low, was not recorded. Several of the issues could have been rectified using electronic questionnaires. This would have allowed the participants to enlarge the question font size so that patients who required reading glasses may have been able to have more autonomy when completing the questionnaires. The questionnaires could also have been linked to electronic health records enabling doctors to review the questionnaire responses.

In 2019, after this work had been completed, The Christie NHS Foundation Trust, a large tertiary cancer center in the United Kingdom, launched its electronic patient-reported outcome measures (ePROMs) service ‘MyChristie-MyHealth,’ integrating ePROMs questionnaires into routine clinical practice (36). This service was initially rolled out to patients with lung cancers, and the adapted-REQUITE lung questionnaire was integrated into the ePROMs platform. The EQ-5D quality of life questionnaire was also added to the questionnaire (37). A study conducted at The Christie by Crockett et al., including 1,480 patients with lung cancer who completed ePROMs questionnaires between January 2019 and December 2020, adds to the evidence supporting the feasibility and practicality of incorporating the REQUITE questionnaire in routine clinical practice (38). This service has been expanded beyond lung cancer to multiple disease sites. At the time of writing this paper, it has enrolled over ten thousand patients with more than thirty-five thousand completed electronic questionnaires featuring the direct lay translation of CTCAE. Both patients and clinicians have responded positively to this service due to its benefits in enhancing care (39).

In recent years, the routine adoption of PROM services has increased worldwide, with a growing emphasis on enabling remote or electronic completion (40–43). This effort effectively bridges the gap between the benefits identified in landmark randomized controlled trials and the application in real-world clinical practice. The benefits around improved consultation, symptom control and clinic efficacy have been realized, as many PROM services are designed to align with clinic appointments. An anticipated advantage identified that has yet to be fully realized involves the ability of PROMs to trigger alerts for clinicians when there is a change in patients’ symptoms from baseline. This could lead to early patient reviews or prompt referrals to urgent services, increasing overall survival. This service is presently being developed at The Christie (36). Therefore, it is of importance that PRO tools facilitate consistent and high-quality symptom reporting, given its increased role in modern healthcare.

As this is the first study to investigate the concordance of these two PRO tools, there is a need to further validate the study findings. Future studies should address the limitations identified in this study, including the inclusion of larger sample sizes accounting for socioeconomic status. It is recommended to conduct longitudinal studies that evaluate PROs at various time points, starting before treatment and continuing through the follow-up period after treatment. This approach provides valuable insights into how patients’ symptoms and quality of life evolve over time.

Additionally, challenges remain for clinicians to establish if a change in patients’ symptoms is caused by the disease progression, adverse events related to treatment, or an exacerbation of their existing comorbidity. Hence, further research is required to assess the impact of patients’ comorbidities on PROs to improve the quality of symptom reporting.

## 5 Conclusion

The adapted-REQUITE questionnaire has shown a better correlation to clinician-reported outcomes and higher patient preference than the PRO-CTCAE^®^. The results of this study have led to the adoption of the adapted-REQUITE questionnaire for patients with lung cancer in routine clinical practice at our institution.

## Data availability statement

The original contributions presented in the study are included in the article/**Supplementary Material**. Further inquiries can be directed to the corresponding author.

## Ethics statement

The studies involving humans were approved by the Health Research Authority, London – City and East Research Ethics Committee, and The Christie Research and Development Division. The studies were conducted in accordance with the local legislation and institutional requirements. The participants provided their written informed consent to participate in this study.

## Author contributions

TJ: Data curation, Formal analysis, Investigation, Methodology, Project administration, Writing – original draft, Writing – review & editing. TN: Visualization, Writing – original draft, Writing – review & editing. IF-W: Visualization, Writing – original draft, Writing – review & editing. RC: Investigation, Resources, Writing – review & editing. JC: Investigation, Resources, Writing – review & editing. MH: Investigation, Resources, Writing – review & editing. HM: Formal analysis, Methodology, Writing – review & editing. PT: Investigation, Resources, Writing – review & editing. DW: Investigation, Resources, Writing – review & editing. CF-F: Conceptualization, Investigation, Methodology, Project administration, Supervision, Validation, Writing – review & editing.
