# Supplementary material for: A study demonstrating users’ preference for the adapted-REQUITE patient-reported outcome questionnaire over PRO-CTCAE® in patients with lung cancer
Source: Front Oncol. 2024 Apr 10;14:1328871. doi: 10.3389/fonc.2024.1328871 (PMC11039780; doi:10.3389/fonc.2024.1328871)

Supplementary Material

Supplementary Table 1 Clinician reported CTCAE symptom grades.

|  | CTCAE grades, n (%) | | | | |
| --- | --- | --- | --- | --- | --- |
|  | 0 | 1 | 2 | 3 | 4 |
| Performance Status* | 24 (38.1) | 26 (41.3) | 8 (12.7) | 5 (7.9) | 0 (0) |
| Dysphagia | 54 (85.7) | 4 (6.3) | 5 (7.9) | 0 (0) | 0 (0) |
| Chest pain | 56 (88.9) | 3 (4.8) | 3 (4.8) | 1 (1.6) | 0 (0) |
| Shortness of Breath | 22 (34.9) | 26 (41.3) | 12 (19.0) | 2 (3.2) | 1 (1.6) |
| Cough | 27 (42.9) | 28 (44.4) | 8 (12.7) | 0 (0) | 0 (0) |
| Haemoptysis | 62 (98.4) | 1 (1.6) | 0 (0) | 0 (0) | 0 (0) |
| Reduced appetite | 33 (52.4) | 18 (28.6) | 12 (19.0) | 0 (0) | 0 (0) |
| Fatigue | 20 (31.7) | 30 (47.6) | 12 (19.0) | 1 (1.6) | 1. (0) |

*Performance status is graded according to the Eastern Cooperative Oncology Group (ECOG) performance status. Abbreviations: CTCAE, Common Terminology Criteria for Adverse Events

Supplementary Table 2 Patient-reported adapted-REQUITE symptom grades.

|  | REQUITE grades, n (%) | | | | |  |
| --- | --- | --- | --- | --- | --- | --- |
|  | 0 | 1 | 2 | 3 | 4 | |
| Performance Status | 25 (39.7) | 21 (33.3) | 13 (20.6) | 4 (6.4) | 0 (0) | |
| Dysphagia | 54 (85.7) | 3 (4.8) | 6 (9.5) | 0 (0) | 0 (0) | |
| Chest pain | 54 (85.7) | 5 (7.9) | 2 (3.2) | 2 (3.2) | 0 (0) | |
| Shortness of Breath | 21 (33.3) | 22 (34.9) | 18 (28.6) | 2 (3.2) | 1 (1.6) | |
| Cough | 27 (42.9) | 27 (42.9) | 9 (14.3) | 0 (0) | 0 (0) | |
| Haemoptysis | 61 (96.8) | 1 (1.6) | 1 (1.6) | 0 (0) | 0 (0) | |
| Reduced appetite | 39 (61.9) | 17 (27.0) | 7 (11.1) | 0 (0) | 0 (0) | |
| Fatigue | 18 (28.6) | 36 (57.1) | 7 (11.1) | 2 (3.2) | 0 (0) | |

Supplementary Table 3 Patient reported PRO-CTCAE® symptom grades.

|  | PRO-CTCAE® grades, n (%) | | | | |
| --- | --- | --- | --- | --- | --- |
|  | 0 | 1 | 2 | 3 | 4 |
| Dysphagia severity | 51 (81.0) | 7 (11.1) | 2 (3.2) | 3 (4.8) | 0 (0) |
| Chest pain frequency | 50 (79.4) | 5 (7.9) | 3 (4.8) | 3 (4.8) | 2 (3.2) |
| Chest pain severity | 50 (79.4) | 5 (7.9) | 3 (4.8) | 3 (4.8) | 2 (3.2) |
| Chest pain’s impact on ADLs | 53 (84.1) | 3 (4.8) | 3 (4.8) | 1 (1.6) | 3 (4.8) |
| Shortness of breath severity | 22 (34.9) | 22 (34.9) | 10 (15.9) | 6 (9.5) | 3 (4.8) |
| Shortness of breath’s impact on ADLs | 27 (42.9) | 21 (33.3) | 7 (11.1) | 6 (9.5) | 2 (3.2) |
| Cough severity | 31 (49.2) | 18 (28.6) | 11 (17.5) | 3 (4.8) | 0 (0) |
| Cough’s impact on ADLs | 51 (81.0) | 9 (14.3) | 2 (3.2) | 1 (1.6) | 0 (0) |
| Reduced appetite severity | 38 (60.3) | 14 (22.2) | 7 (11.1) | 3 (4.8) | 1 (1.6) |
| Reduced appetite’s impact on ADLs | 50 (79.4) | 11 (17.5) | 1 (1.6) | 1 (1.6) | 0 (0) |
| Fatigue severity | 19 (30.2) | 23 (36.5) | 11 (17.5) | 9 (14.3) | 1 (1.6) |
| Fatigue’s impact on ADLs | 24 (38.1) | 25 (39.7) | 4 (6.4) | 8 (12.7) | 2 (3.2) |

Abbreviations: ADL, Activities of Daily Living; PRO-CTCAE®*,* Patient Reported Outcomes-Common Terminology Criteria for Adverse Events

Supplementary Table 4 Grading difference between CTCAE and adapted-REQUITE (n=63).

|  | Grade difference, n (%) | | | | | |
| --- | --- | --- | --- | --- | --- | --- |
|  | Patient graded symptom higher | | Agreement | Clinician graded symptom higher | | |
|  | +2 grades | +1 grade | 0 | | +1 grade | +2 grade |
| Performance Status | 0 (0) | 6 (9.5) | 53 (84.1) | | 4 (6.3) | 0 (0) |
| Dysphagia | 0 (0) | 1 (1.6) | 62 (98.4) | | 0 (0) | 0 (0) |
| Chest pain | 0 (0) | 3 (4.8) | 60 (95.2) | | 0 (0) | 0 (0) |
| Shortness of Breath | 0 (0) | 12 (19.0) | 44 (69.8) | | 6 (9.5) | 1 (1.6) |
| Cough | 1 (1.6) | 5 (7.9) | 52 (82.5) | | 4 (6.3) | 1 (1.6) |
| Haemoptysis | 1 (1.6) | 1 (1.6) | 60 (95.2) | | 1 (1.6) | 0 (0) |
| Reduced appetite | 0 (0) | 3 (4.8) | 48 (76.2) | | 10 (15.9) | 2 (3.2) |
| Fatigue | 0 (0) | 6 (9.5) | 50 (79.4) | | 7 (11.1) | 0 (0) |

Abbreviations: CTCAE, Common Terminology Criteria for Adverse Events

Supplementary Table 5 Grading difference between CTCAE and PRO-CTCAE® (n=63).

|  | Grade difference, n (%) | | | | | | |
| --- | --- | --- | --- | --- | --- | --- | --- |
|  | Patient graded symptom higher | | | Agreement | Clinician graded symptom higher | | |
|  | +2 grades | +1 grade | 0 | | | +1 grade | +2 grade |
| Dysphagia | 1 (1.6) | 6 (9.5) | 54 (85.7) | | | 2 (3.2) | 0 (0) |
| Chest pain | 3 (4.8) | 7 (11.1) | 53 (84.1) | | | 0 (0) | 0 (0) |
| Shortness of Breath | 4 (6.3) | 12 (19.0) | 40 (63.5) | | | 0 (0) | 0 (0) |
| Cough | 1 (1.6) | 11 (17.5) | 44 (69.8) | | | 6 (9.5) | 1 (1.6) |
| Haemoptysis |  |  |  | | |  |  |
| Reduced appetite | 3 (4.8) | 8 (12.7) | 38 (60.3) | | | 13 (20.6) | 1 (1.6) |
| Fatigue | 5 (7.9) | 18 (28.6) | 31 (49.2) | | | 9 (14.3) | 0 (0) |

This table features PRO-CTCAE® assessing severity of symptoms only, not assessing impact of symptom on activities of daily living. Abbreviations: CTCAE, Common Terminology Criteria for Adverse Events

Supplementary Table 6 Reasons given for preferring adapted-REQUITE over PRO-CTCAE® (n=34).

| Reason | N (%) |
| --- | --- |
| Questionnaire was more descriptive and less subjective | 22 (64.7) |
| Questionnaire was easier to complete | 10 (29.4) |
| Questionnaire gave a better description of symptoms | 1 (2.9) |
| Required help with the other questionnaire | 1 (2.9) |

Supplementary Table 7 Reasons given for preferring PRO-CTCAE® over adapted-REQUITE (n=14).

| Reason | N (%) |
| --- | --- |
| Questionnaire was easier to complete | 8 (57.1) |
| Questionnaire required less reading to complete | 4 (28.6) |
| Borderline for some grades in the REQUITE tool | 1 (7.1) |
| Questionnaire gave a better description of symptoms | 1 (7.1) |


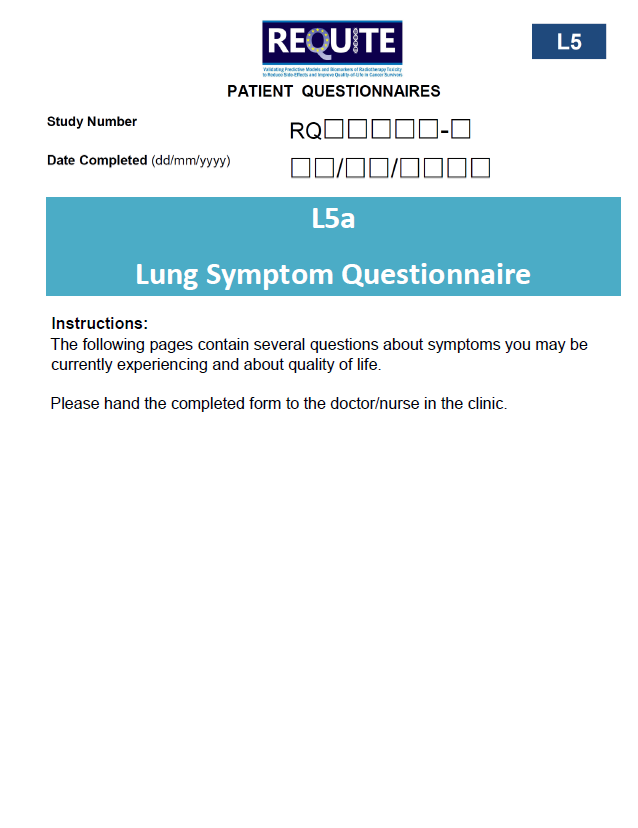


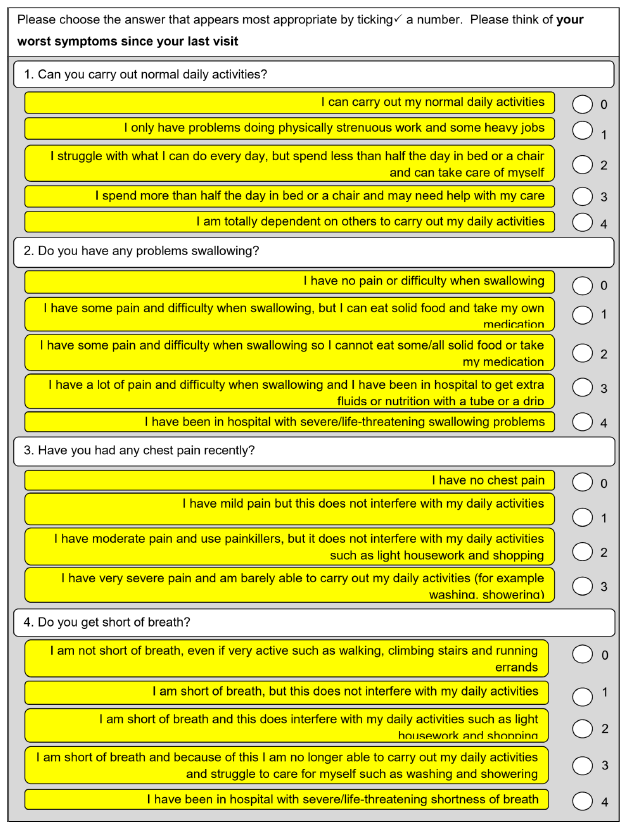


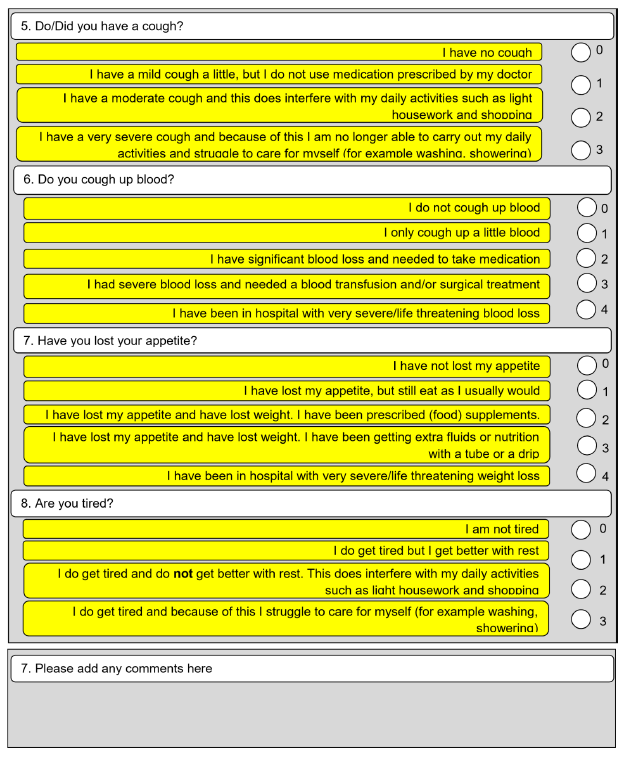


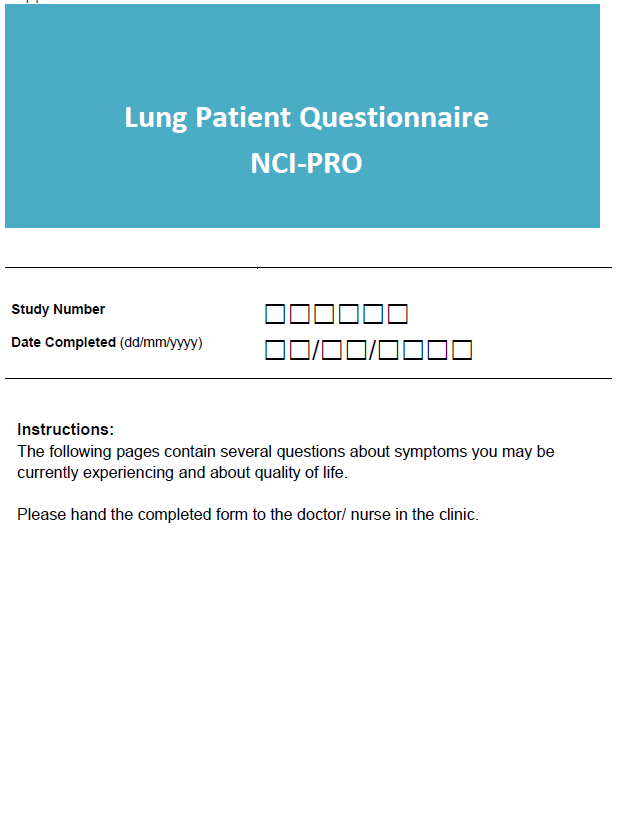


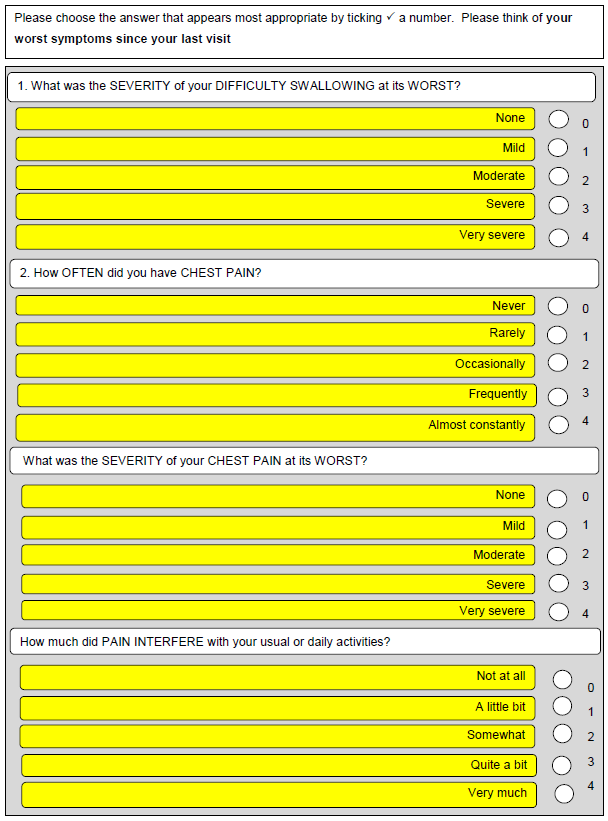

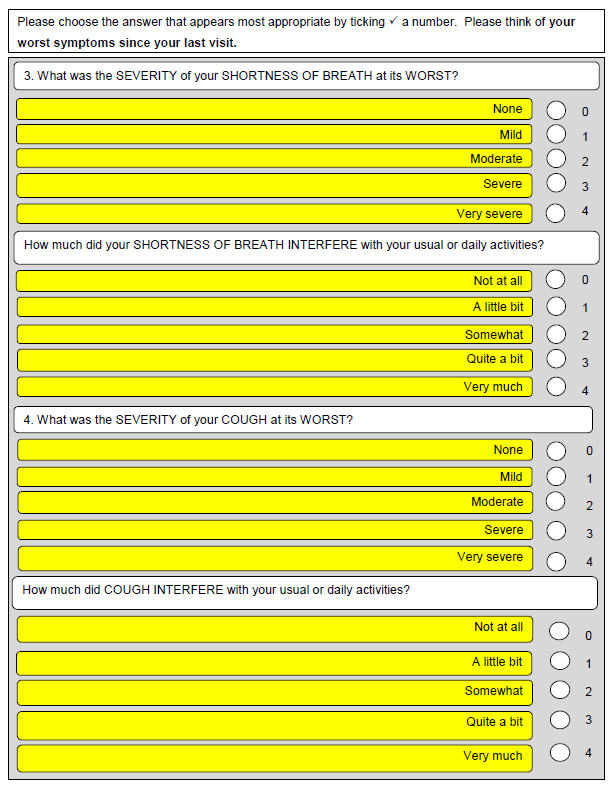


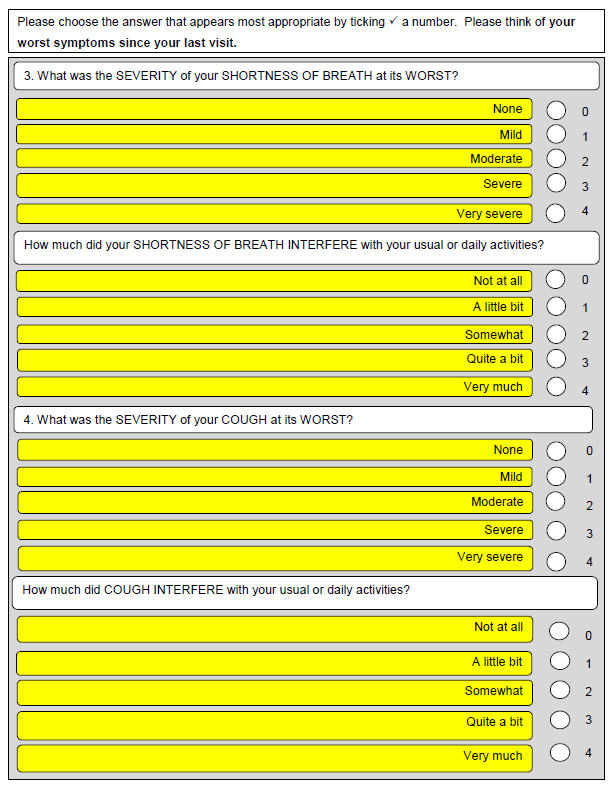


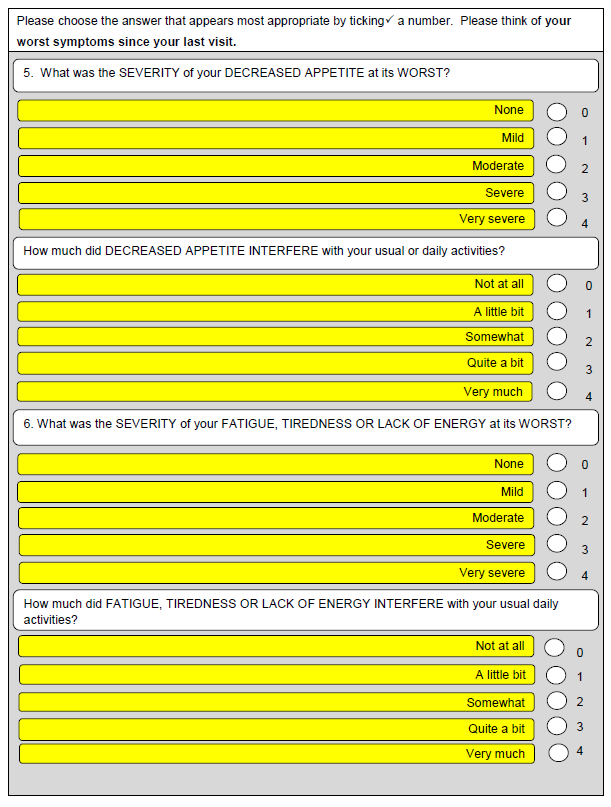


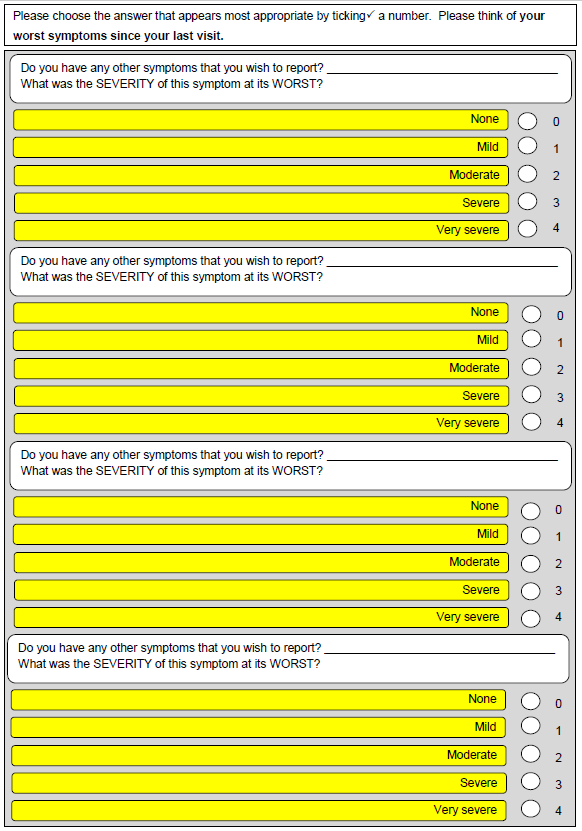

Supplement: Supplementary file 1 [file DataSheet_1.docx]
